# Heart Rate Variability (HRV) in Patients with Sleep Apnea and COPD: A Comprehensive Analysis

**DOI:** 10.3390/jcm14134630

**Published:** 2025-06-30

**Authors:** Andreea Zabara-Antal, Radu Crisan-Dabija, Raluca-Ioana Arcana, Oana Elena Melinte, Adriana-Loredana Pintilie, Ionela Alina Grosu-Creanga, Mihai Lucian Zabara, Antigona Trofor

**Affiliations:** 1 Clinical Hospital of Pulmonary Diseases Iasi, 700116 Iasi, Romania; andreeazabara@yahoo.com (A.Z.-A.); raluca.dospinescu@yahoo.com (R.-I.A.); rohozneanuoana@yahoo.com (O.E.M.); pintilie.adriana.loredana.mg6.7@gmail.com (A.-L.P.); ionela.grossu@yahoo.com (I.A.G.-C.); atrofor@yahoo.com (A.T.); 2 Faculty of General Medicine, Grigore T. Popa University of Medicine and Pharmacy Iasi, Doctoral Student, 700115 Iasi, Romania; 3 Faculty of General Medicine, Pulmonary Department, Grigore T. Popa University of Medicine and Pharmacy Iasi, 700115 Iasi, Romania; 4 Faculty of General Medicine, Surgery Department, Grigore T. Popa University of Medicine and Pharmacy Iasi, 16 Universitatii, 700115 Iasi, Romania; mihai-lucian.zabara@umfiasi.ro; 5 Department of General Surgery and Liver Transplantation, St. Spiridon University Hospital Iasi, 700115 Iasi, Romania

**Keywords:** HRV, OSA, COPD, sleep study, overlap syndrome, polygraphy, Continuous Positive Airway Pressure (CPAP)

## Abstract

Background: Obstructive sleep apnea (OSA) and chronic obstructive pulmonary disease (COPD) are prevalent conditions with overlapping clinical features and shared consequences on autonomic function. Heart rate variability (HRV), a non-invasive biomarker of autonomic nervous system activity, may offer diagnostic, prognostic, and therapeutic insights in this patient population. Methods: A comprehensive literature review was conducted using PubMed, Google Scholar, and MEDLINE to identify peer-reviewed English-language studies published between January 2015 and December 2024. Studies were included if they evaluated HRV parameters in individuals with OSA, COPD, or overlap syndrome, explored HRV as a marker of disease severity or progression. A total of 239 studies were identified; after screening, 41 met the inclusion criteria. Results: The analysis revealed consistent evidence linking reduced HRV with both OSA and COPD severity. HRV alterations were more pronounced in overlap syndrome, reflecting synergistic autonomic dysfunction. HRV showed potential in differentiating disease stages, predicting cardiovascular risk, and evaluating treatment efficacy, particularly for CPAP therapy in OSA. Short-term HRV was particularly sensitive to autonomic changes, while long-term recordings helped track disease progression. Emerging evidence supports the use of HRV derived from wearable technologies as a viable screening tool for health and wellness. Conclusion: HRV is a valuable, non-invasive tool for assessing autonomic dysfunction in OSA, COPD, and their overlap. It offers significant potential for early diagnosis, disease monitoring, and treatment evaluation. Integrating HRV into clinical practice, could enhance diagnostic efficiency, reduce healthcare burden, and improve outcomes in high-risk respiratory populations. Furthermore, longitudinal studies are warranted to standardise HRV thresholds and validate their use in routine screening protocols.

## 1. Introduction

In 1996, the Task Force of the European Society of Cardiology and the North American Society of Pacing and Electrophysiology published the first comprehensive guidelines for measuring heart rate variability (HRV). Over the subsequent decades, research on HRV expanded significantly, exploring its relationship with various physiological and pathological conditions, including stress, anxiety, chronic disorders, and sleep-related issues [1]. The cardiac autonomic nervous system is divided into the parasympathetic and sympathetic branches, which are controlled by centres located in the midbrain, hypothalamus, medulla, and pons. The sympathetic nerves originate from the intermediolateral column of the first to fifth thoracic spinal segments and consist of two components based on their anatomical position: extrinsic and intrinsic. The extrinsic sympathetic nerves originate from the superior cervical ganglia and the cervicothoracic ganglia, which connect with several cervical nerves (C1–C3, C7–C8) and thoracic nerves (T1–T2). Additionally, the thoracic ganglia contain the cell bodies of most postganglionic sympathetic neurons, whose axons form the superior, middle, and inferior cardiac nerves. These nerves terminate on the surface of the heart, where they form a network of nerve fibres and ganglionic plexuses, collectively creating the intrinsic sympathetic nerves of the heart. Then, noradrenaline is released from the synapses, and once released, it triggers the sympathetic nerves and excites the sinoatrial and atrioventricular nodes, thereby increasing the heart rate, accelerating the repolarization of the myocytes, and strengthening the myocardium’s ability to contract [2].

Heart rate variability (HRV) is considered a non-invasive biomarker for assessing the body’s autonomic regulatory physiological pathways. HRV is an emergent property of the body’s regulatory systems, arising from the interplay of multiple factors, including the balance between sympathetic and parasympathetic nervous system activity, blood pressure regulation mechanisms (e.g., the baroreceptor reflex), intrinsic cardiac pacemaker activity, gas exchange, and respiratory function. A healthy heart exhibits complex, nonlinear oscillations in its beat-to-beat intervals, which enable it to adapt to varying environmental demands, such as physical activity, stress, or rest. However, pathophysiological changes can disrupt this complexity, diminishing the heart’s ability to cope with challenges [2,3]. It measures the fluctuations in the time intervals between consecutive heartbeats, specifically the RR intervals extracted from electrocardiographic (ECG) recordings, which are directly controlled by sympathetic and parasympathetic activity [2]. Each RR interval represents the elapsed time between successive R peaks of the QRS complex on an ECG, reflecting the dynamic balance between sympathetic and parasympathetic activity. Circadian variation in HRV offers additional insights into cardiac autonomic function. A study analysing 24 individuals demonstrated that healthy young adults (approximately 35 years old) experience a decline in cardiac rhythm complexity, which is less pronounced at night than during the day. In contrast, individuals aged 68 and older exhibit a loss of this complexity, with no discernible circadian variation [3].

Standard methods of HRV data acquisition include various pieces of equipment, such as heart rate monitors with one or more channels, which process ECG signals via an analogue-to-digital converter for analysis. For clinical purposes, 24 h Holter systems and cardiac belts remain the gold standard for HRV monitoring, as they enable long-term recordings that can predict clinical outcomes [4]. Short-term HRV recordings (greater than five minutes) and ultra-short HRV recordings (10–30 s) are also used to assess cardiac events. Advances in wearable technology now enable both long-term and short-term HRV monitoring during various activities, including exercise, sleep, and rest. Data collected via wearable devices provide practical and cost-effective alternatives to traditional clinical ECG recordings, with meta-analyses demonstrating minimal error when compared to clinical measurements. The length of the HRV recording plays a pivotal role in capturing the nuances of autonomic function. More extended recording periods provide a more comprehensive representation of minimal fluctuations and responses to diverse stimuli [5].

HRV analysis also plays a key role in assessing sympathetic hyper-reactivity and shifts in the autonomic nervous system. The standard and most common HRV measurement techniques are confined to linear methods, such as time-domain and frequency-domain analyses. Time-domain analysis involves calculating statistical measures from the intervals between RR peaks of successive QRS complexes in a continuous ECG recording, evaluating the variability of the RR interval of the sinus rhythm using statistical and geometric methods. Commonly used indices include the mean standard deviation of NN intervals (SDNN), the mean standard deviation of the index (SDNNI), and the HRV triangular index. These measures are used to estimate overall heart rate variability (HRV) and reflect sympathetic nerve tension; smaller values are associated with higher levels of sympathetic nerve tension. Clinically, low HRV indicates increased sympathetic activity, which is associated with a poor prognosis for cardiovascular pathology. Additionally, the overall standard deviation reflects the balance between sympathetic and parasympathetic nerve tension. Frequency-domain analysis, on the other hand, decomposes RR intervals into their physiological rhythms, producing a power spectrum calculated using parametric or non-parametric methods. The spectrum curve obtained through power spectral density analysis provides fundamental information on how variance is distributed as a function of frequency. In current practice, spectral analysis performed on a 5 min ECG recording identifies three main spectral components: very low frequencies (≤0.04 Hz), low frequencies (0.04–0.15 Hz), and high frequencies (0.15–0.4 Hz). Sympathetic activity is assessed using low frequencies, while the ratio of low to high frequencies correlates with the balance between sympathetic and parasympathetic nerve activities. These analyses can serve as indicators of cardiovascular diseases such as myocardial infarction, hypertension, and heart failure [6]. These analyses of HRV are stable and straightforward methods of assessing sympathetic nerve activity. However, they cannot extract key information from the complex interactions of hemodynamic, electrophysiological, and moral variables, as well as autonomic and central nervous regulations, known as nonlinear phenomena, which can be analysed using nonlinear methods. Using the Poincaré plot, it is possible to plot every RR interval against the prior interval, create a scatter plot, and extract the correlations between successive RR intervals over different time scales [2].

Sleep disorders generally affect HRV. This parameter is altered during all sleep stages in patients with sleep conditions, with lower values observed during non-REM stages compared to REM sleep. Insomnia and OSA seem to correlate frequently with HRV alteration [3].

## 2. Methods and Study Design

We conducted a comprehensive literature review using three major databases: PubMed, Google Scholar, and MEDLINE. The objective was to identify studies and publications examining the effects of heart rate variability (HRV) in patients diagnosed with obstructive sleep apnea (OSA) and chronic obstructive pulmonary disease (COPD). The literature review spanned the period from January 2015 to December 2024, encompassing recent and relevant HRV analysis within this clinical context. A total of 239 studies were initially identified through database searches. After title and abstract screening, 121 full-text articles were assessed for eligibility. Of these, 43 studies met all inclusion criteria and were included in the final review. Studies were included if they investigated HRV parameters in individuals with OSA, COPD, and overlap syndrome, explored HRV as a marker of autonomic dysfunction, disease severity, or progression, and employed standard methods of HRV analysis, and were published in English in peer-reviewed journals. Several studies were excluded, including those that included case reports, animal studies, conference abstracts, and articles that did not provide a direct correlation between HRV and either OSA or COPD. A key focus of this review was to explore the physiological changes related to HRV in this patient population and to understand how these changes might correlate with disease progression, severity, and outcomes. Another crucial aspect of this analysis is to explain the process of data acquisition and the analysis of HRV parameters in order to understand the results better. An important aim of this paper was to evaluate the literature search employing critical terms, including “Sleep”, “Sleep study”, “Polygraphy”, “Polysomnography”, “OSA”, “HRV in OSA patients,” “HRV analysis”, “HRV in COPD”, “Continuous Positive Airway Pressure” and combinations of these terms to ensure a thorough exploration of the association between the two conditions. We aimed to identify studies that evaluated HRV as a non-invasive biomarker for autonomic dysfunction, particularly within the context of OSA, COPD, and the combined condition known as overlap syndrome. Data extraction emphasized HRV metrics (such as time-domain, frequency domain, and nonlinear measures) and their correlation with sleep apnea and chronic obstructive pulmonary disease). To facilitate comparison across studies and highlight trends, a comprehensive summary is provided in Appendix A Table A1 which outlines key study characteristics, HRV parameters assessed, sample sizes, and main findings. Most studies reported a reduction in overall HRV (e.g., lower SDNN and RMSSD) in association with increasing disease severity, with sympathetic dominance (e.g., elevated LF/HF ratios) particularly pronounced in overlap syndrome. Interventional studies evaluating CPAP, mandibular advancement devices, and sleep surgery have also demonstrated improvements in HRV metrics, particularly in the time-domain and high-frequency components.

## 3. Obstructive Sleep Apnea Syndrome (OSAS)—A Modern Challenge

Humans still dedicate a quarter of their existence to sleeping, even if the need for slumber is decreasing nowadays, considering the evolution of society. Sleep quantity and architecture significantly impact life quality. Currently, approximately 90 sleep disorders are described in the literature. All of these have effects at an individual level as well as at a societal level. This includes reduced productivity and the incurring of high medical costs. OSA is the most involved in morbidity and mortality. Obstructive sleep apnea is not only highly prevalent but is also independently associated with significant morbidity and mortality: untreated severe OSA increases all-cause mortality by ~1.9-fold and cardiovascular mortality by ~2.6-fold [7]. Obstructive sleep apnea triggers persistent sympathetic nervous system activation, promoting oxidative stress, inflammation, endothelial dysfunction, and autonomic imbalance. Longitudinal studies have demonstrated that patients with OSA are at heightened risk of developing hypertension, arrhythmias, myocardial infarction, and heart failure. HRV analysis provides a non-invasive window into these autonomic disturbances and may serve as an early indicator of cardiovascular risk in this population [8].

The combination of chronic obstructive lung disease and sleep apnea defines the “overlap syndrome,” and the combination of pathophysiological processes is more complex [5]. Therefore, understanding this complex histopathology is critical for early identification and tailored interventions to reduce morbidity and improve life quality [9].

Obstructive sleep apnea (OSA) is a highly prevalent condition with significant implications for the patients with chronic lung disease outcomes. It is estimated to affect approximately 9–38% of the population, with a higher prevalence rate observed in men (13–33%) compared to women (6–19%). Several risk factors contribute to OSA development, including obesity (a major contributor), craniofacial and oro-pharyngeal abnormalities, or smoking [10]. OSA is characterized by partial to total collapse of the upper airways, leading to transient reductions in airflow, large intrathoracic pressure swings, and intermittent hypoxia [5]. These repeated episodes of hypoxemia and hypercapnia trigger a cytokine-mediated inflammatory cascade, resulting in elevated levels of catecholamines, oxidative stress, and low-grade systemic inflammation. This inflammatory response plays a critical role in the pathophysiological changes associated with OSA and contributes to its widespread health consequences. Notably, OSA-induced physiological changes are strongly linked to an increased risk of cardiovascular morbidity and mortality. Patients with OSA are at greater risk of developing other conditions such as heart failure, stroke, and coronary artery disease.

## 4. Heart Rate Variability—A Screening Tool for OSAS?

The mechanisms underlying cardiovascular impairment in OSA are complex and multifactorial, involving sympathetic overactivity, endothelial dysfunction, inflammation, oxidative stress, and the development of atherosclerotic plaques in the cerebral vasculature, coupled with altered cerebral blood flow regulation, which impairs the development of vascular dementia [8]. Among these, sympathetic overactivity, particularly in moderate to severe forms of OSA, has been identified as a significant factor driving adverse cardiovascular outcomes. Previous research has stated that long-term HRV is reduced in OSA patients, even during the daytime, and an increase in OSA severity is linked with shorter RR intervals. Respiratory events, such as apneas that last longer, can generate a greater decrease in the RR interval after the event. Regardless of sex, in the case of ultra short-term HRV, when more prolonged apneas and hypopneas occur, the average RR interval during the respiratory event is longer. In contrast, the post-event RR interval is shorter. The analysis of the parameters used to evaluate HRV (standard deviation of RR intervals, root mean square of the successive differences, and proportion of successive RR intervals differing by more than 50 ms) showed an increase, raising the apnea and hypopnea duration. After an event lasting longer than 20–30 s or more than 30 s, HRV variables are greater compared to apneas that last 10–20 s. Shorter apneas are correlated with a higher difference between within-event and post-event HRV parameters. In men, these findings are more pronounced [10]. Innovative diagnostic approaches have also highlighted the potential of heart rate variability (HRV) as a predictor for OSA. Lao et al. conducted a study involving 63 adults who underwent 24-h ECG Holter monitoring in conjunction with polysomnography to assess the sensitivity and specificity of Holter-derived respiratory waveforms for SOSA screening. The study revealed a sensitivity of 90% and a specificity of 82.6%, emphasizing the utility of HRV in identifying OSA [11]. Furthermore, in frequency-domain analysis, an increase in the LF/HF ratio was observed in patients with severe forms of OSA, highlighting the potential application of HRV metrics for severity assessment. Sleep fragmentation, another hallmark of OSA, is closely associated with cardiovascular and autonomic dysfunction. Pronounced sleep fragmentation has been correlated with higher nocturnal HRV and subsequent sympathovagal imbalance, further exacerbating the cardiovascular risks associated with OSA [12]. When discussing daytime vigilance, patients with sleep apnea often exhibit impaired vigilance and excessive daytime sleepiness due to recurrent hypoxic episodes during sleep. However, overnight recordings of heart rate variability (HRV) do not appear to be strongly associated with poor vigilance in these patients. This suggests that overnight HRV measurements may not fully capture the physiological stress or autonomic dysregulation caused by obstructive sleep apnea (OSA) that contributes to daytime impairment. Instead, short-term HRV analyses, which reflect acute changes in vagal modulation and interbeat variability, may provide a more accurate indicator of impaired vigilance and its underlying mechanisms in this population. The findings highlight the limitations of overnight HRV as a “static” measure and advocate for dynamic, short-term HRV assessments to capture physiological stress [13].

Nowadays, 1 billion people are diagnosed with OSA all over the world [13]. Even so, over 80% of patients with obstructive sleep apnea syndrome (OSAS) remain undiagnosed. This suggests that they either lack highly suggestive symptoms or overlook them [14] Ultimately, although management is straightforward, access to sleep testing remains limited. The identification of a screening tool for early detection is indispensable. The current literature is attempting to qualify HRV as a marker for early diagnosis of OSAS [15]

Currently, a significant proportion of research efforts is directed toward identifying means of early diagnosis that are generally applicable across various diseases. This is especially critical when dealing with a condition that is constantly evolving. Equally important is the association of sleep apnea with other comorbidities. Cardiovascular pathology, diabetes mellitus, and urological disorders become more challenging to manage when sleep apnea is a concurrent factor. Specific blood pressure patterns, elevated morning blood pressure values, and treatment-refractory hypertension often prompt cardiologists to investigate an underlying cause. Like a particular element, it is interesting to mention that patients with excessive sleepiness have a higher risk of cardiovascular diseases than less sleepy patients with similar severity of OSA or OSA absence. The prevalence of OSA associated with excessive daytime sleepiness is 3% in women and 5% in men in the general population. Talking about people with cardiovascular or metabolic conditions, the prevalence is significantly higher [16].

Poor glycaemic control in a patient with known and optimally treated diabetes or the emergence of a new diabetes case may conceal underlying sleep apnea. Also, respiratory comorbidities are known to be hard to manage when SAOS exist.

## 5. Obstructive Sleep Apnea and Chronic Obstructive Pulmonary Disease (COPD)—A Concerning Association

Chronic obstructive pulmonary disease (COPD) is a chronic inflammatory lung condition characterized by persistent and progressive airflow limitation. It is the third leading cause of mortality worldwide, with a prevalence of 13.1%. The airflow limitation in COPD is primarily a consequence of exposure to inhaled toxic particles and gases, particularly from smoking. This exposure results in a range of pathological changes, including small airway inflammation, obstructive bronchiolitis, and emphysema, all of which contribute to airway destruction and impaired lung function. As a chronic illness, COPD has a systemic impact and can influence the autonomic nervous system (ANS). Cardiac autonomic function, in particular, can be evaluated non-invasively through heart rate variability (HRV), which serves as a measure of autonomic nervous system (ANS) activity. A systematic review and meta-analysis of patients with COPD revealed an overall decrease in HRV parameters. This decrease reflects a significant disruption in the coupling between the heart’s intrinsic pacemaker and extrinsic mechanisms governed by the autonomic nervous system. In patients with COPD, cardiac control appears to shift towards a parasympathetic predominance compared to healthy individuals [17]. The reduction in HRV observed in patients with COPD has been associated with disease severity. Studies suggest that HRV indices can serve as markers for the progression and severity of COPD, offering potential for monitoring and risk stratification [18]. Moreover, circadian patterns of HRV reveal further insights into autonomic dysfunction in COPD. Research indicates that patients with COPD and/or heart failure experience a more pronounced decline in HRV at night compared to during the day, suggesting a disrupted day-night variation in autonomic control [19]. Studies show that half of the patients with COPD experience poor sleep quality. Nocturnal hypoxemia, due to hypoventilation and altered ventilation/perfusion ratios, leads to changes in sleep architecture. Just one night of sleep deprivation is sufficient to impact functional parameters. However, COPD patients do not typically report highly suggestive symptoms of sleep apnea syndrome. Symptoms are often attributed to the underlying chronic disease. Nevertheless, the coexistence of both conditions leads to challenges in managing each illness. Identifying a screening tool that allows for the early diagnosis of sleep apnea in patients known to have COPD is essential.

The co-occurrence of COPD and OSA, commonly referred to as overlap syndrome, is found in approximately 1% of adults. It was first described in 1985. This is when, although there was limited knowledge of OSA, the American Thoracic Society stated research priorities for this condition. There were some screening strategy recommendations in order to identify OSA in COPD patients with chronic stable hypercapnia [16].

Most studies report a high prevalence of OSA in COPD patients, ranging from approximately 55.7% overall in broader COPD cohorts to as high as 65.9% in patients with moderate-to-severe COPD undergoing sleep laboratory evaluation [15,16].

This dual diagnosis presents complex combined pathophysiological effects that have profound implications for patients’ health. While some physiological effects of COPD can be protective against OSA, other changes are strongly correlated with an increased risk of developing the condition. Among the protective factors, tracheal traction resulting from lung hyperinflation caused by emphysema plays a significant role in protecting the airways. This mechanical effect reduces upper airway collapsibility and decreases the severity of obstructive sleep apnea [19]. However, despite these protective mechanisms, many patients with COPD remain at elevated risk for OSA due to systemic inflammation, impaired respiratory mechanics, and other factors associated with their condition. In addition to the physiological interplay between COPD and OSA, sleep quality is profoundly affected in patients with overlap syndrome. Sleep becomes fragmented, with a reduction in the duration and quality of both slow-wave and REM sleep. Interestingly, while apneas are more likely to occur during REM sleep, the diminished occurrence of this phase in overlap syndrome patients may paradoxically reduce the frequency of these episodes [20]. Nonetheless, this disruption of restorative sleep phases contributes to significant reductions in overall quality of life, increased fatigue, and worsened health outcomes. The chronic inflammation observed in COPD smoker individuals, having a higher body mass index, has been strongly associated with an increased risk of developing obstructive sleep apnea (OSA). This risk is particularly pronounced due to the combined effects of systemic inflammation and altered upper airway mechanics. In addition, hypoxaemia caused by COPD, which results from underlying conditions such as emphysema and pulmonary hypertension, introduces physiological changes that exacerbate respiratory challenges. When coupled with the upper airway collapse characteristic of apneic episodes, these factors can rapidly lead to significant drops in blood oxygen levels [21]. The intermittent hypoxia associated with OSA further compounds these issues by impairing gas exchange, which has been shown to result in a higher incidence of daytime hypercapnia in patients with overlap syndrome compared to those with COPD alone [22]. This combination of conditions places a substantial burden on the patient’s respiratory system and overall health. Furthermore, the risk of exacerbations is notably higher in patients with overlap syndrome. Studies indicate that these individuals face a 1.7-fold greater likelihood of recurrent acute exacerbations compared to patients with COPD alone. This heightened risk underscores the complex interplay between OSA and COPD in overlap syndrome and the need for tailored clinical interventions [23]. Interestingly, variations in heart rate may serve as a predictive marker for severe acute exacerbations in chronic lung diseases. Specifically, lower short-term variability in heart rate has been suggested as a potential indicator of such exacerbations [22]. In addition to the linear methods of assessing HRV, a study involving 297 patients with COPD and moderate-to-severe OSA used sample entropy to distinguish changes in overnight pulse rate variability (PRV) and evaluate long-term changes in irregularity. It was found that overnight HRV, evaluated using entropy, exhibited a significantly more irregular pattern in individuals diagnosed with overlap syndrome compared to patients with OSA alone. This suggests that the cumulative effect of both diseases increases the disorganization of pulse rate during sleep. Another important conclusion from this study is that when analysing frequency bands using linear methods, no statistically significant differences were found between patients with COPD and those with COPD and OSA. However, the analysis using sample entropy could be useful in assessing cardiovascular impairment in COPD patients due to the presence of concomitant sleep apneas [24]. When comparing individuals diagnosed with COPD or OSA to those presenting with both conditions, commonly known as overlap syndrome, HRV frequency domain analysis revealed that high-frequency power was significantly diminished, while low-frequency power was significantly increased. These findings suggest that individuals with overlap have higher sympathetic modulation of heart rate compared to those with OSA or COPD alone [25]. Normally, with each breathing cycle, the heart rate increases during inspiration and decreases during expiration due to the control of autonomic nervous system activity. When both COPD and OSA are present, and respiratory sinus arrhythmia manoeuvres are performed, patients exhibit increased sympathetic modulation, as indicated by increased low-frequency power and a higher low-to-high frequency ratio, coupled with decreased high-frequency power —a pattern opposite to what is expected during these manoeuvres. Additionally, in individuals with both OSA and COPD, heart rate variability exhibited a worse complexity response compared to patients diagnosed with COPD alone [26]. The Six-Minute Walk Test (6MWT) remains a simple, widely available test used in clinical practice, and HRV undergoes physiological changes that influence autonomic nervous system regulation. Zangandro et al. evaluated HRV changes in individuals with OSA-COPD and discovered a strong correlation between HRV indices and nocturnal desaturation. Specifically, they found that the longer the time spent with oxygen saturation below 90%, the greater the parasympathetic modulation during walking [27].

Recently, research has focused on identifying data indicating that smokers exhibit reduced heart rate variability (HRV) compared to non-smokers, also establishing a correlation between the smoking intensity and the degree of impairment of this cardiac parameter. Additionally, efforts directed towards to demonstrate the improvement of HRV following smoking cessation. A study conducted on 4751 adults, comparing smokers to non-smokers, established that individuals in the former category recorded lower HRV values, reflected in the standard deviation of NN intervals (SDNN) and high-frequency power (HF), which indicate parasympathetic activity. Not only the patient’s status as a chronic smoker impact the cardiac parameter, although the number of pack-years correlates with the degree of HRV impairment. Interestingly, recent smoking is also capable of temporarily reducing HRV, particularly the component related to vagal regulation [28].

A thorough and comprehensive review published in the International Journal of Cardiology evaluates consistent evidence with significant biological impact regarding the effects of smoking on heart rate variability (HRV). It concludes that the majority of studies implicate disruptions in the functioning of the autonomic nervous system, both in active and passive smoking [29]. Bodin et al., in an article published in *Psychosomatic Medicine*, report similar findings. They assessed 149 healthy individuals. In smokers, HF-HRV was reduced by 0.31 ms^2^ during periods of recent smoking compared to periods without smoking. The 24 h impairment was significantly more pronounced in smokers compared to non-smokers [30].

If we consider the clear damage of HRV in patients with sleep apnea and this additional deterioration due to their status as smokers, we are dealing with an immense risk of cardiovascular complications stemming from two sources.

Obesity is the primary risk factor for the development of sleep apnea syndrome. At the same time, it is also a consequence. Through sleep deprivation, the metabolism of leptin and ghrelin is affected, leading to altered appetite and an increase in body mass index. Thus, the patient experiences a vicious cycle of obesity, which is both a cause and an effect of the condition. On the other hand, patients with GOLD stage 3–4 COPD usually have a reduced BMI. However, paradoxically, nearly 40% of patients with overlap condition exhibit metabolic syndrome [15]. Talking about the risk factors for OSA in COPD patients, we can also mention the following: the rostral fluid shift in the supine position that contributes to OSA severity, tracheal displacement that may contribute to the maintenance of pharyngeal patency in dogs, normal subjects, and OSA patients (43–45 A) smoking, associated with an increase in sleep latency and arousal frequency as well as a decrease in deep sleep stages [31]. SAOS exacerbates the severity of COPD and complicates its management. Exacerbation of the underlying disease become more frequent and more severe. Effective sleep apnea management can improve COPD management.

Related to the clinical presentation in patients with overlap syndrome, compared to those with only one of the conditions, there is a more pronounced level of daytime hypoxia and nocturnal hypercapnia. Regarding the last condition, there are different opinions. Some articles only involve the combination of overweight and altered lung function. Other studies have shown that PaO_2_ contributed to 38%, FEV1 to 15%, and body weight to 12% in the gas alteration development. The literature suggests that the presence of sleep apnea impacts cardiorespiratory fitness in patients with COPD. The severity class of OSA correlates with the degree of impairment [32].

To resume, SAOS exacerbates the severity of COPD and complicates its management. The exacerbation of the underlying disease becomes more frequent and severe. Effective sleep apnea management can improve COPD management. Identifying an element that qualifies as a biomarker for early detection, which is associated with the severity class and plays a role in predicting the likelihood of progression, is helpful [33].

The reduction in heart rate variability (HRV) is a negative prognostic factor, both in the general population and even more so in individuals with cardiac pathology. The clinical significance of changes in this parameter has been associated with determining the risk of sudden death due to arrhythmic episodes. HRV alteration correlates with the presence of sleep apnea syndrome, and the degree of impairment of this parameter is directly proportional to the severity class of obstructive sleep apnea syndrome (OSAS). Thus, it is a promising candidate for serving as a marker of sleep disorders and their comorbidities [34].

There are emerging technologies designed to enhance heart rate variability (HRV) analysis. As a result, the diagnostic accuracy for obstructive sleep apnea syndrome (OSAS) is being refined. This also enhances the detection rate of cardiovascular risk. Furthermore, the timely identification and correction of markers associated with severity class have a positive impact on quality of life while reducing mortality and morbidity [9] (see Table 1).

**Table 1:** Summarizes key heart rate variability (HRV) parameters across three patient populations: obstructive sleep apnea (OSA), chronic obstructive pulmonary disease (COPD), and their overlap syndrome.

Patient Group	Time-Domain Measures (e.g., SDNN, RMSSD)	Frequency-Domain Measures (e.g., LF, HF, LF/HF)	Typical HRV Findings	Clinical Implications	HRV Measurement Description
OSA	↓ SDNN, ↓ RMSSD (more reduced with apnea duration >30 s)	↑ LF, ↓ HF, ↑ LF/HF (especially in severe OSA)	Sympathetic overactivity, HRV reduction persists during daytime	Higher cardiovascular risk, may aid in OSA diagnosis	SDNN (Standard Deviation of NN intervals) reflects overall HRV; RMSSD (Root Mean Square of Successive Differences) reflects parasympathetic activity.
COPD	↓ SDNN, variable RMSSD (parasympathetic predominance)	↓ LF and HF, altered circadian variation	Blunted autonomic response, reduced HRV linked with disease severity	Useful for disease monitoring, associated with poor sleep and prognosis	SDNN reflects global autonomic variability; RMSSD indicates short-term parasympathetic modulation, often preserved or variably reduced in COPD.
Overlap Syndrome	↓↓ SDNN, ↓ RMSSD (greater reduction than either condition alone)	↑↑ LF/HF, ↓ HF, ↑ LF (enhanced sympathetic dominance)	Additive autonomic dysfunction, increased pulse irregularity (entropy)	Predicts exacerbations, aids in identifying overlap; HRV complexity worsens	SDNN and RMSSD are both significantly reduced; this suggests compounded autonomic dysregulation due to dual pathophysiology.

↓ = Mild to moderate decrease. ↓↓ = Marked or more pronounced decrease. ↑ = Mild to moderate increase. ↑↑ = Marked or more pronounced increase.

## 6. Obstructive Sleep Apnea, COPD and Heart Rate Variability: Does Therapy Help?

Continuous positive airway pressure (CPAP) therapy is widely recognized as the gold standard treatment for patients diagnosed with obstructive sleep apnea, as it maintains the patency of the upper airway and prevents its collapse during sleep, which is a primary cause of apneas and hypopneas. These repeated episodes of airway obstruction lead to intermittent hypoxemia and fragmented sleep, resulting in significant activation of the sympathetic nervous system. By effectively addressing the episodes of hypoxemia and restoring standard breathing patterns during sleep, CPAP therapy has a beneficial influence on HRV. It modulates autonomic function by reducing sympathetic nervous system activity and promoting parasympathetic dominance. During CPAP titration, a significant change in HRV parameters has been observed in most studies, indicating a restoration of balance between sympathetic and parasympathetic activity, particularly notable in males with severe forms of obstructive sleep apnea [35]. Several studies have demonstrated that a single night of continuous positive airway pressure (CPAP) therapy results in a relative improvement in autonomic system activity, as measured by HRV indices [36]. In patients with mild to severe obstructive sleep apnea (OSA), a noticeable shift toward reduced heart rate variability (HRV) is observed one month after initiating continuous positive airway pressure (CPAP) therapy, particularly in those with severe OSA, defined by an apnea-hypopnea index (AHI) exceeding 30 events per hour. This finding suggests that the effectiveness of CPAP therapy in modulating HRV may depend on the severity and frequency of obstructive events prior to treatment [37]. Two months of CPAP therapy and aerobic exercise training can improve cardiac autonomic dysfunction, as measured by assessing HRV parameters. Regarding mild to moderate forms of obstructive sleep apnea (OSA), when CPAP therapy is not tolerated, mandibular advancement devices can be used as an alternative treatment. After a minimum of three months of usage, a recorded decrease in the apnea-hypopnea index by 18 events per hour has been observed. Additionally, HRV parameter analysis reveals an increased low-to-high frequency ratio and low-frequency power, accompanied by a reduction in high-frequency power. This suggests that mandibular advancement devices used for treatment correlate with changes in HRV, indicating improved cardiac autonomic adaptability [38]. Lastly, several upper airway interventions can reduce the collapsibility present during apneas, including tonsillectomy, uvulopalatopharyngoplasty, expansion sphincter pharyngoplasty, lateral pharyngoplasty, tongue-base resection, and other combinations. When comparing HRV indicators of patients using mandibular advancement devices with those who underwent surgical intervention after three months of treatment, in patients with a range of respiratory events from 5 to 40, there is a similarly significant increase in average normal-to-normal intervals in time-domain analysis. In frequency-domain analysis, the LF/HF ratio decreased in both groups, while normalized high-frequency power increased significantly, in contrast to normalized low-frequency power. These findings suggest that sleep surgery and mandibular advancement devices are equally effective treatments for OSA in terms of improving cardiac autonomic activity [39]. In a study of nineteen stable moderate-to-severe COPD patients, non-invasive ventilation with bi-level positive airway pressure (BiPAP) was compared to spontaneous breathing. The results showed lower low-frequency (LF) power, a reduced LF/HF ratio, and increased high-frequency (HF) power compared to a control group of healthy age-matched individuals. During BiPAP, high-frequency HRV was significantly reduced. At the same time, low-frequency HRV increased, suggesting that non-invasive ventilation is associated with decreased cardiac vagal activity in stable moderate-to-severe COPD patients [40,41].

Alternatively, regarding treatment for COPD, a Phase 4 study that included 34 patients found that only indacaterol administration led to an increased heart rate compared with the placebo. Additionally, neither long-acting β_2_-agonists nor muscarinic antagonists were found to have deleterious effects on cardiovascular autonomic control. Additional data suggest that sympathetic stimulation, achieved through the use of short-acting and long-acting β-sympathomimetic agonists, causes tachycardia. After adjusting HRV metrics according to the mean heart rate period or heart rate, it was found that the concurrent use of β-sympathomimetic agonists and muscarinic antagonists was associated with reduced HRV in 39 individuals diagnosed with COPD. Furthermore, in well-phenotyped COPD patients, decreased HRV metrics are correlated with increased breathlessness, worsening COPD symptoms, and declining health status, as assessed by their individual-specific questionnaires [41,42].

Recent papers suggest that the restoration of HRV following the initiation of continuous positive airway pressure in OSA patients seems to be slower in smokers. CPAP improves HRV metrics within weeks in non-smokers, while the other category exhibits a delayed response. Only marginal response is observed after three months of correctly conducted treatment. This is because of the persistent sympathetic over-activation and reduced vagal tone, which are not fully mitigated by CPAP alone [43].

## 7. Conclusions

Heart rate variability (HRV) is well established as a marker of autonomic dysfunction in individuals diagnosed with COPD or OSA, and their co-existence, widely known as overlap syndrome, has a profound impact on autonomic regulation due to their combined pathophysiologic effects.

Firstly, in patients diagnosed with OSA alone, HRV can be considered a predictor of disease severity. The longer the recorded apneas, the shorter the RR intervals, and after an event occurs, an increased HRV variability is observed. These consequences extend into the daytime, with long-term HRV recordings showing a sustained reduction. HRV can also be used as a diagnostic tool for OSA screening; in Holter-derived HRV assessments of OSA, the diagnosis was confirmed with 90% sensitivity and 82.6% specificity.

Secondly, similar findings are encountered in individuals diagnosed with COPD, with a significant decrease in HRV recorded, suggesting a weakened connection between the heart’s pacemaker and autonomic control mechanisms. As in OSA, HRV indices can serve as markers of COPD severity, making them useful for monitoring disease progression and risk stratification.

Additionally, during the night, COPD patients exhibit a greater decline in HRV metrics compared to during the day. When these two pathophysiological effects combine, they result in more profound cardiovascular and respiratory impacts in overlap syndrome. HRV frequency analysis has revealed higher sympathetic activity in overlap syndrome, and HRV response complexity worsens compared to COPD alone. Furthermore, the analysis of lower short-term HRV may help predict severe exacerbations, with the risk of acute exacerbation being 1.7 times higher in the presence of overlap syndrome.

Various treatments for overlap syndrome have demonstrated effects on HRV, with improvements observed even during CPAP titration. Most of the time, after one month of CPAP therapy, an improvement in autonomic function was recorded. It is worth noting that in more severe forms of OSA, HRV modulation is more pronounced with the use of CPAP therapy.

This review highlights the crucial role of heart rate variability (HRV) as a promising screening tool for identifying obstructive sleep apnea (OSAS) in patients with chronic obstructive pulmonary disease (COPD), a population at increased risk. The consistent association between reduced HRV and OSA severity, coupled with its sensitivity to autonomic dysfunction, a hallmark of both COPD and OSAS, positions HRV as a non-invasive, accessible, and cost-effective parameter for early identification. Furthermore, the prognostic value of HRV in predicting cardiovascular complications, which are amplified in the COPD–OSAS overlap, enhances its clinical utility. While further longitudinal studies are warranted to standardise HRV thresholds and validate its specificity in this subgroup, integrating HRV into routine screening protocols could significantly improve diagnostic rates, optimise therapeutic interventions, and mitigate the compounded morbidity of these coexisting conditions.

**Discussions**

Based on the findings of this review, HRV offers several key clinical utilities that can be utilised during routine care. In the setting of primary care or outpatient pulmonary clinics, HRV can be utilised as a screening tool to identify individuals at risk of OSAS or COPD-related autonomic dysfunction. With 80% sensitivity and specificity, wearable devices, such as wrist-worn monitors, enable convenient home-based HRV measurement, potentially reducing the burden on specialised sleep laboratories.

In individuals with a prior established diagnosis of COPD or OSAS, serial HRV analysis may help detect declining HRV metrics that could signify worsening disease control, especially in the case of overlap syndrome. While polysomnography remains the gold standard for diagnosing sleep disorders, it is resource-intensive and often delayed due to access barriers. HRV monitoring, particularly via commercially available wearable technologies, offers a cost-effective adjunct or alternative in specific contexts.

The ability to conduct repeated, longitudinal assessments at a fraction of the cost of laboratory-based studies may improve diagnostic coverage and health system efficiency, particularly in underserved or resource-limited populations. To operationalise HRV in clinical practice, standardisation of data acquisition protocols (e.g., time-domain vs. frequency-domain metrics, optimal recording durations) and threshold values for diagnostic interpretation is crucial. Integration into electronic health records and decision-support tools will further facilitate clinician uptake.

Although a meta-analysis of heart rate variability (HRV) outcomes across studies investigating obstructive sleep apnea (OSA), chronic obstructive pulmonary disease (COPD), and their overlap syndrome would offer important quantitative insights, this review identified substantial heterogeneity in study design and methodology that precluded such an analysis. Differences were noted in HRV acquisition protocols (e.g., duration, device type, and analysis methods), and the types of HRV metrics reported (e.g., time-domain, frequency-domain, nonlinear indices). Due to this variability, a pooled statistical synthesis was deemed inappropriate.

In conclusion, HRV holds substantial translational value in the diagnostic and management continuum of OSA and COPD. As wearable technologies mature and validation studies proliferate, HRV is poised to become a cornerstone of personalized, autonomic-guided care.

## Data Availability

All data relevant to the review are included within the article. No new datasets were generated.

## Abbreviations

6MWT	Six-Minute Walk Test
AHI	Apnea-Hypopnea Index
ANS	Autonomic Nervous System
BiPAP	Bilevel Positive Airway Pressure
BMI	Body Mass Index
COPD	Chronic Obstructive Pulmonary Disease
CPAP	Continuous Positive Airway Pressure
ECG	Electrocardiogram
HF	High Frequency
HRV	Heart Rate Variability
LF	Low Frequency
LF/HF	Low Frequency to High Frequency Ratio
NN intervals	Normal-to-Normal Intervals
OSA	Obstructive Sleep Apnea
OSAS	Obstructive Sleep Apnea Syndrome
PRV	Pulse Rate Variability
REM	Rapid Eye Movement
SDNN	Standard Deviation of NN Intervals
SDNNI	Standard Deviation of the NN Interval Index

## Appendix A

**Table A1:** Study characteristics, HRV metrics, and key findings from empirical investigations in obstructive sleep apnea, chronic obstructive pulmonary disease, and overlap syndrome.

Author (Year)	Sample Size (n)	Population	HRV Parameters	Key Findings
Shaffer & Ginsburg, 2017 [1]	404–4752 (pooled)	Healthy individuals (HRV normative datasets)	SDNN, RMSSD, LF, HF, LF/HF, entropy	Defined normal HRV ranges and methodology recommendations.
Hietakoste et al., 2020 [11]	758	Suspected OSA patients from PSG	Ultra-short-term HRV, RR intervals	Longer apneas/hypopneas → increased post-event HRV.
Romero et al., 2021 [13]	81	OSA patients (mild/moderate vs. severe)	VLF_nu_, LF_nu_, HF_nu_	Non-REM HRV best reflects OSA severity; severe OSA → higher LF_nu_.
Sattar et al., 2024 [3]	40	Idiopathic REM behavior disorder vs. controls	Time-/frequency-domain HRV (resp-adjusted)	HRV altered in REM; emphasizes need for respiratory correction.
Lao et al., 2021 [12]	63	Adults undergoing Holter + PSG for OSA	Time-domain, respiratory waveform HRV	HRV screening: 90% sensitivity, 82.6% specificity for OSA.
Borghi Silva et al., 2008 [42]	19	COPD patients (BiPAP vs. spontaneous breathing)	LF, HF, LF/HF	BiPAP → increased sympathetic, decreased vagal activity.
Murgia et al., 2019 [27]	4751	General population (CHRIS study)	SDNN, HF power	HRV inversely correlated with smoking intensity.
Bodin et al., 2017 [28]	149	Healthy smokers vs. non-smokers	HF-HRV (24-h recordings)	Recent smoking lowers HF-HRV; long-term suppression observed.
Hietakoste et al., 2024 [17]	70	Suspected OSA patients	Short-term vs. overnight HRV (SDNN, RMSSD)	Short-term HRV better predicts impaired daytime vigilance.
Serrão et al., 2020 [18]	41	COPD patients	SDNN, RMSSD, entropy, DFA	Reduced HRV complexity correlates with higher GOLD class and symptoms.
Ma et al., 2023 [20]	50	COPD, HF, and healthy controls	Time, frequency, and complexity (day-night HRV)	Flattened circadian HRV and reduced complexity in chronic cardiopulmonary disease.
Park et al., 2008 [31]	105	OSA patients stratified by AHI severity	SDNN, RMSSD, LF, HF, LF/HF	HRV decreases with OSA severity; LF/HF increases in severe OSA.
Álvarez et al., 2019 [33]	87	COPD, OSA, and overlap syndrome patients	Time, frequency, entropy	HRV lowest in overlap group; strongest sympathetic activation noted.
Taranto Montemurro et al., 2016 [34]	38	COPD vs. OSA-COPD overlap patients	Time-domain, LF/HF ratio	Cardiac sympathetic hyperreactivity highest in overlap syndrome.
Camargo et al., 2023 [35]	31	COPD patients with/without OSA	RSA maneuver, time-domain HRV	OSA worsens cardiac autonomic response to RSA in COPD.
Zangrando et al., 2018 [36]	35	COPD patients with/without OSA	Time-domain HRV, 6MWT analysis	Nocturnal desaturation linked to greater parasympathetic activity during walking.
Shin et al., 2023 [37]	58	OSA patients before and after positive airway pressure	SDNN, RMSSD	CPAP acutely reduced sympathetic tone and improved HRV.
Efazati et al., 2020 [38]	62	Moderate and severe OSA patients (CPAP during PSG)	RMSSD, LF, HF, LF/HF	CPAP reduced LF/HF and increased vagal HRV indices after one night.
Grzęda Hałon et al., 2023 [39]	52	OSA patients using CPAP	SDNN, RMSSD, HF power	CPAP improved parasympathetic tone and HRV complexity over time.
Ucak et al., 2024 [40]	47	OSA patients using mandibular advancement device	SDNN, LF/HF ratio	Improved HRV modulation following oral appliance therapy.
Lee et al., 2019 [41]	36	OSA patients: surgery vs. mandibular advancement device	LF, HF, LF/HF	Both groups improved; surgery showed greater LF/HF shift.
Park et al., 2022 [43]	30	COPD patients using wearable monitor	SDNN, RMSSD	Lower HRV associated with more severe COPD classification.
